# Supplementary material for: Effects of adding dual-task or sport-specific task constrains to jump-landing tests on biomechanical parameters related to injury risk factors in team sports: a systematic review
Source: PeerJ. 2024 Aug 13;12:e17720. doi: 10.7717/peerj.17720 (PMC11328837; doi:10.7717/peerj.17720)
Supplement: Supplemental Information 2 [file peerj-12-17720-s002.docx]

## Rationale for Conducting the Systematic Review/Meta-Analysis

The rationale behind conducting this systematic review stems from the multifactorial nature of performance in team sports, which requires athletes to constantly adapt to unpredictable and changing game situations through a combination of conditional, coordinative, and cognitive factors. This adaptation involves executing complex motor actions while processing large amounts of information and simultaneously responding to multiple stimuli, highlighting the significance of Dual-Task (DT) scenarios where athletes perform cognitive and motor tasks concurrently. The existing literature acknowledges the challenges posed by DT on performance due to the limited information processing capacity of the central nervous system, which affects the allocation of attention and the ability to perform multiple tasks. Despite the extensive research on DT effects and the consensus on its impact on task performance, a gap exists in systematically reviewing how incorporating DT during jump-landing tests affects biomechanical variables related to lower-limb injury risk in team sports. This review aims to fill that gap by examining the influence of DT on these biomechanical variables, contributing to a deeper understanding of injury risk factors in the context of real-sport situations.

## Contribution to Knowledge

This systematic review contributes to the existing body of knowledge by specifically focusing on the effects of incorporating DT during jump-landing tests on biomechanical variables related to lower-limb injury risk in team sports. Prior reviews have explored the effect of task anticipation on movement mechanics and the influence of DT in individuals with ACL injuries, but none have specifically analyzed the impact of DT during jump-landing actions. By doing so, this review provides new insights into how cognitive, coordinative, or decision-making tasks included in jump-landing tests can alter biomechanical variables, suggesting a higher risk of injury under such conditions. This contribution is significant in light of previous reports and meta-analyses, as it highlights the need for jump-landing tests that more accurately simulate the specific demands of competition, including cognitive and coordinative challenges.

The review also identifies a lack of studies focusing on comparative analyses between limbs and recording errors in secondary tasks, suggesting directions for future research. This is crucial for developing a comprehensive understanding of the dynamics of motor and cognitive processes in sports, with implications for injury prevention and recurrence reduction. The findings emphasize the limitations of traditional jump-landing tests and advocate for the development and validation of sport-specific tests that incorporate DT and constraints to assess athletes’ functional status and risk factors more effectively. Such advancements would enable physical trainers and sports rehabilitators to implement more precise and safer intervention strategies, ultimately enhancing the quality of athlete care and rehabilitation in the context of team sports.
